# Effects of source-sink regulation and nodal position of the main crop on the sprouting of regenerated buds and grain yield of ratoon rice

**DOI:** 10.3389/fpls.2023.1043354

**Published:** 2023-03-27

**Authors:** Aibin He, Min Jiang, Lixiao Nie, Jianguo Man, Shaobing Peng

**Affiliations:** ^1^ MOA Key Laboratory of Crop Ecophysiology and Farming System in the Middle Reaches of the Yangtze River, College of Plant Science and Technology, Huazhong Agricultural University, Wuhan, Hubei, China; ^2^ Research Center for Physiology and Ecology and Green Cultivation of Tropical Crops, College of Tropical Crops, Hainan University, Haikou, Hainan, China

**Keywords:** ratoon rice, leaf-cutting, spikelet-thinning, cytokinin, regeneration rate

## Abstract

Ratoon rice (Oryza sativa L.) is the production of a second season rice that utilizes the dormant buds surviving on the stubble left behind after the harvest of the main crop. However, the sprouting mechanism of regenerated buds at separate nodes is rarely reported. Field experiments were conducted to examine the effects of leaf-cutting and spikelet thinning on the sprouting of regenerated buds at the separate node, the contributions of regenerated panicles at the separate node to the total grain yield in the ratoon crop, and the associated mechanism. The results showed that the contribution of separate node yields to the total grain yield in the ratoon crop was D2 (panicles regenerated from the 2^nd^ node from the top) >D3 (panicles regenerated from the 3^rd^ node from the top) >D4 (panicles regenerated from the lower nodes below the 3^rd^ node), and the contribution of D2 and D3 made up approximately 80% of the total yield in the ratoon crop. In addition, the effect of leaf-cutting treatment and spikelet-thinning treatment on the grain yield of ratoon season was mainly realized by regulating the relative contribution rate of D2 and D4 grain yield to the total yield of ratoon season. Further analysis indicated that the sprouting of regenerated buds at the D2 node was mainly affected by the content of CTK, while D3 was mainly regulated by GAs and CTK, and D4 was mainly regulated by ABA and CTK. However, only the CTK content in stems and buds was positively correlated with single bud length and bud number at each nodes. These results indicated that CTK might be the main signal regulating the sprouting of regenerated buds and the grain yield at separate nodes, which might change the transport of assimilates to stems and buds.

## Highlights

1. Spikelet-thinning treatment significantly increased the yield contribution of D2 to the total grain yield in ratoon season while markedly reduced the yield contribution of D4, while leaf-cutting treatment reduced the yield contribution of D2 to the total grain yield in ratoon season, increased the yield contribution of D4.
2. Analysis revealed that leaf-cutting and spikelet-thinning treatments mainly changed the CTK content in stems and buds at each node of the ratoon crop.
3. The sprout of regenerated buds at the D2 node was mainly affected by the content of CTK.

## 1 Introduction

Ratoon rice refers to the rice cropping system that utilizes the dormant buds surviving on the rice stubble after the harvest of the main crop and allows them to germinate into regenerated panicles under suitable cultivation conditions, which is suitable for planting in areas where the annual accumulation of temperature and light resources is considerably greater than that required for single-cropping rice but not enough for double-season rice (Chen et al., 2018; He et al., 2019). Ratoon rice has drawn much attention and has been widely practiced by farmers because of its advantages in labor, seed, water, pesticides and seedbed savings, and the selection of varieties with high regeneration rates (Wang et al., 2019). For example, the planting area of ratoon rice was progressively increased from 26,700 ha in 2013 to 213,300 ha in 2020 in Hubei Province, China (Wang et al., 2021). It was predicted that a total of 13.28 million ha could be planted with ratoon rice without the reduction of current planting area of double-season rice in China by using the Maxent model (Yu et al., 2021). Ratoon rice has developed rapidly and becomes one of the major cropping systems to improve rice production in central and southern China. It was reported that ratoon rice exhibited a significantly higher net energy ratio and benefit-to-cost ratio and substantially lower yield-scaled global warming potential (GWP) than middle-season rice and double-season rice cropping systems (Yuan et al., 2019). It was reported that when the input of resources and labor cost was reduced by 50%, the grain yield of ratoon crop could reach 60% of that of the main season (Wang et al., 2020). Furthermore, the nutrition, cooking and eating quality of rice in the ratoon season were better than that those in the main season (Alizadeh and Habibi, 2016). In addition, the economic benefit of ratoon rice was significantly higher than that of double-season rice, which mainly resulted from the input of labor, seeds, fertilizer, and insecticide of ratoon being reduced by 28.5%, 51.9%, 31.5 and 41.1%, respectively (Liang et al., 2016; Sen and Bond, 2017). Meanwhile, the annual yield for ratoon rice system was 25.3% higher than that for traditional single season rice production (Shen et al., 2020). Therefore, the high and stable yield of ratoon rice is of great significance to ensuring food security and improving planting efficiency in China.

The yield of the ratoon crop is mainly affected by the number of effective panicles or regeneration rate (Chen et al., 2018). Promoting the sprouting and panicle formation of regenerated buds is the key to ensuring the high yield of ratoon crop (Lin et al., 2015). Many studies have been conducted to improve the sprouting of regenerated buds through various agronomic practices. It was reported that the application of bud-promoting fertilizer significantly increased the effective panicles in the ratoon crop compared with non-bud fertilizer application (Santos et al., 2003; Yu et al., 2017). Studies have indicated that the high stubble height of the main season improved the sprouting of regenerated buds and the yield of the ratoon crop, which resulted from increased stubble dry weight and non-structural carbohydrate content (Xu et al., 1997; Ren et al., 2006; Yazdpour et al., 2012; He et al., 2019). Harrell et al. (2009) reported that the 20 cm stubble height of the main season increased the number of regenerated buds that regenerated from the basal nodes compared to the 40 cm stubble height. Moreover, flood irrigating immediately after the harvest of the main crops effectively promoted the tillering of the ratoon crop (Mengel and Wilson, 1981). The application of exogenous plant growth regulators significantly improved the sprouting of regenerated buds (Quddus and Pendleton, 1991; Xie et al., 2017). The mechanisms underlying these agronomic practices may break the equilibrium between source and sink and affect the distribution and transport of substances (Vergara et al., 1988; Albacete et al., 2014; Yu et al., 2015).

Plant hormones, as signal substances in plants, play an important role in the sprouting and growth of buds. IAA (indole-3-acetic acid) is synthesized in the apical meristem and transported to basal tissue through polar transport to inhibit lateral bud growth. Previous studies indicated that the growth of axillary buds of gramineous crops is also affected by apical dominance by IAA (Chatfield et al., 2000; Mohapatra, 2011; Xia et al., 2020). Removing apical dominance can break the dormancy of buds and promote their sprouting. Furthermore, previous studies have shown that plant hormones ABA (abscisic acid), CTK (cytokinins), BR (brassinosteroid), GAs (gibberellins), and SLs (strigolactones) are involved in axillary bud or regenerated bud growth and regulation (Chatfield et al., 2000; Sae and Hitoshi, 2001; Cheng et al., 2013; Xu et al., 2020). It was reported that after the harvesting of the main season, a higher IAA content in the stem or a higher ratio of plant hormone (IAA, GA and CTK) to ABA promoted the ratoon crop of regenerated buds (Xiao, 2002). Studies have shown that the content of GA_3_ is closely related to the sprouting of regenerated buds, and the content of GA_3_ increases rapidly with the rapid sprouting of regenerated buds at 20 days after the heading of the main season (Liu et al., 1998; Li and Tang, 2003). Furthermore, it was shown that GAs, iPAs, and BR play a vital role in the sprouting of axillary buds of ratoon rice (Cheng et al., 2003; Xu et al., 2020). In addition, it was clear that IAA and CTK were synergistically or antagonistically involved in regulating tiller or axillary bud growth in rice (Shimizu-Sato and Mori, 2001; Su et al., 2011; Xia et al., 2020), and it was demonstrated that tiller bud growth was regulated by CTK derived from nodes of rice, which was regulated by IAA (Liu et al., 2011). The above-documented literature Indicated some studies about the effect of endogenous hormones on the sprouting of regenerated buds of ratoon rice. However, there are few researches on the relationship between the content of endogenous hormones and the sprouting of regenerated buds at separate nodes of ratoon rice.

Leaf-cutting and spikelet thinning are two commonly used methods to study source-sink relationships in crops (Das et al., 2018). Our previous study indicated that leaf-cutting and spikelet-thinning treatments at the full heading stage of the main crop had various effects on the regeneration ability and yield of ratoon rice, which was associated with the changes in the ratio of grain number to green leaf area, stubble dry weight and non-structural carbohydrates content in the stubble (He et al., 2019). However, the effects of leaf-cutting and spikelet thinning treatments on regenerated buds at the separate node and the yield contribution of the separate node to yield in ratoon crops are unclear. Therefore, the objectives of the present study were (1) to examine the effects of leaf-cutting (cutting the first leaf from the top, and cutting top three leaves) and spikelet thinning (thinning 1/4, 1/2 and 3/4 of spikelets) treatments on growth of regenerated buds at separate nodes and the yield contribution of each node to total yield in ratoon crop, (2) to explore how endogenous hormones regulate the sprouting of regenerated buds at separate nodes, (3) and to regulate better the regeneration ability and grain yield of ratoon crop in real production.

## 2 Materials and methods

### 2.1 Experimental site

Experiments were conducted in Jiupu Village (30°14′N, 115°25′E), Chidong Town, Qichun County, Hubei Province, China, during the rice growing seasons of 2018 and 2019 in two adjacent paddy fields. Before the experiment, two adjacent paddy fields were planted with ratoon rice for many years. The soil backgrounds of the adjacent paddy fields were similar. The pH, total nitrogen (N), available phosphorus, potassium, and organic matter in the upper 20 cm of the soil before rice planting were 5.01, 0.18%, 13.27 mg kg^-1^, 144.90 mg kg^-1^, and 25.33 g kg^-1^, respectively. Fields were plowed, harrowed, and leveled before sowing.

### 2.2 Experimental design

**Figure 1:**
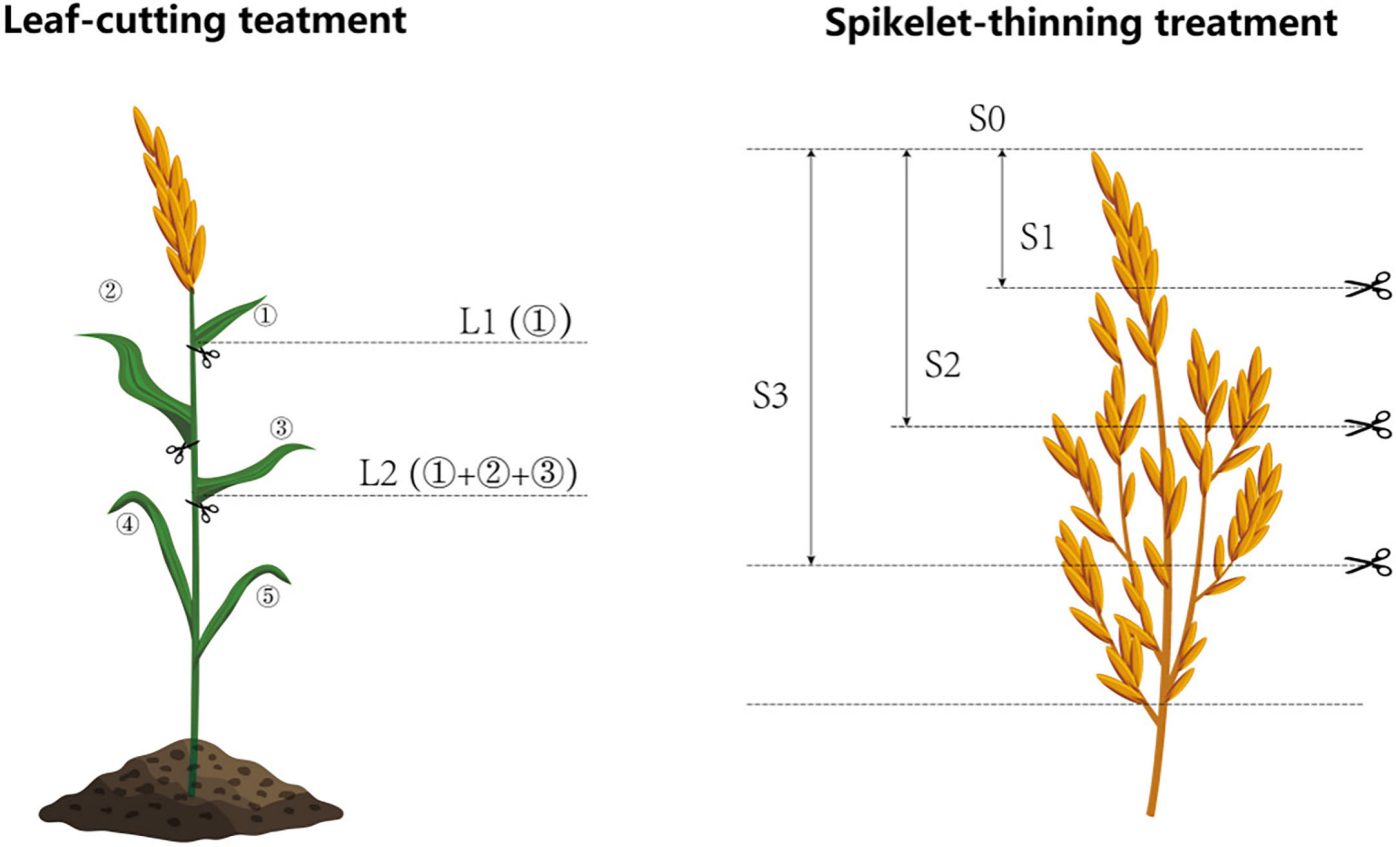
Schematic diagram of the treatment of leaf-cutting and spikelet-thinning. L0 represent the treatment of no leaf-cutting; L1 represent the treatment of cutting top of one leaf; L2 represent the treatment of cutting top of three leaves; S0, S1, S2 and S3 represent the treatment of thinning 0, 1/4, 1/2 and 3/4 spikelets, respectively.

A hybrid rice (*Oryza sativa L.*) cultivar Liangyou6326 (LY6326), the major rice cultivar for ratoon rice in central China, was used for two years. The study was laid out in a randomized complete block design using four replicates (plot size: 3 m × 3 m in 2018, and 3 m × 4 m in 2019). In 2018, three leaf-cutting treatments (L0: no leaf-cutting, L1: cutting the first leaf from the top and L2: cutting top three leaves) and two spikelet-thinning treatments (S0: no spikelet-thinning and S2: thinning one-half of the spikelets) were combined to compose six treatments (L0S0, L0S2, L1S0, L1S2, L2S0, L2S2). However, based on the field experiment in 2018, two more spikelet-thinning treatments were adopted in 2019. In 2019, three leaf-cutting treatments (L0: no leaf-cutting, L1: cutting the first leaf from the top, and L2: cutting the top three leaves) and four spikelet-thinning treatments (S0: no spikelet-thinning, S1: thinning 1/4 the spikelets, S2: thinning one half of the spikelets and S3: thinning 3/4 the spikelets) were combined to compose twelve treatments (L0S0, L0S1, L0S2, L0S3, L1S0, L1S1, L1S2, L1S3, L2S0, L2S1, L2S2, and L2S3) (**Figure 1**). All the treatments were conducted at the full heading stage of the main crop and before pollination, and all plants at each plot was treated accordingly. In the leaf-cutting treatments, the first leaf from the top of the top three leaves was removed from each stem. For the treatments of thinning 1/4, 2/4, and 3/4 of the spikelets, the proportion of thinning spikelets was cut according to the length of the panicle. The growth duration of all treatments in 2018 was reported by He et al. (2019), and the growth duration in 2019 was provided in **Supplementary Table 1**.

Pregerminated seeds were sown in nurseries on March 30 in both 2018 and 2019. The transplanting dates were April 20 in both years. All plots were plowed and puddled before transplanting. Seedlings were transplanted into the paddy soil with a hill spacing of 30 × 13.3 cm, with 2 seedlings per hill. In the main crop, a fertilizer dose of 180:40:100 of N:P:K kg ha^-1^ was applied to all treatments. All of the P, one-third of the N, and half of the K were applied as a basal starter dose, while the residual N was equally split at the middle tillering stage and the panicle initiation stage, and the other 50% of the potassium was top-dressed during the panicle initiation stage. In the ratoon crop, a fertilizer dose of 150:40 of N:K kg ha^-1^ was applied equally to all treatments. Half of the N and all of the potassium were applied 15 days after flowering of the main crop as bud-promoting fertilizer, and the other 50% of the N was applied 3 days after harvest of the main crop. The sources of N, P, and K were urea (46.0% N), calcium superphosphate (12.0% P_2_O_5_), and potassium chloride (60.0% K_2_O), respectively. Weeds, diseases, and insects were intensively controlled throughout the entire growing season in both years.

### 2.3 Data recorded

#### 2.3.1 Weather data

Meteorological data, including maximum and minimum temperature, rainfall, and solar radiation during the duration of whole rice growth, were collected from a weather observatory (AWS800; Campbell Scientific, Inc., USA) installed near the experimental field (the straight-line distance does not exceed 300 m).

#### 2.3.2 Investigation of the sprouting of regenerated buds

Six hills with uniform growth were sampled from each plot to measure the number and length of regenerated buds and the fresh weight of a single regenerated bud at the maturity stage of the main crop (MS). After washing, the regenerated buds from each node were stripped out, and the length of each bud, the total number of buds, and the total fresh weight of buds from the six sampled hills were recorded in the D2 node (panicles regenerated from 2^nd^ node from the top), D3 node (panicles regenerated from the 3^rd^ node from the top), and D4 node (panicles regenerated from the lower nodes below the 3^rd^ node) (Dong et al., 2017). The length and number of regenerated buds at each node of all effective panicles on 6 hills were measured and calculated. Then, the length of a single regenerated bud, the number of regenerated buds per m^2,^ and the fresh weight of a single regenerated bud from each node or the whole plant were calculated. The panicles, stems, leaves, buds, and stubble were dried at 80°C to a constant weight.

#### 2.3.3 Grain yield and regeneration rate

At the maturity stage of the main season, twelve hills (0.5 m^2^) with similar panicle numbers and heading dates were sampled to calculate the yield and effective panicles. At the maturity stage of the ratoon crop, 12 hills (0.5 m^2^) with similar panicle numbers and heading dates were sampled to calculate the yield and effective panicles at separate nodes. And the cutting height of the main crop was 40 cm. Similarly, the grain yields in D2, D3, and D4 nodes were calculated as described by Dong et al. (2017). In all nodes, the panicle number was counted in each plot to determine the panicle number per m^2^. The regeneration rate (RR) at D2, D3, and D4 was calculated as the ratio of “the number of effective panicles in D2, D3 and D4” to “the number of effective panicles in the main season”.


(1) $$\begin{array}{l} \text{Regeneration rate }(\text{RR})\\ =\text{the number of panicles in D}2,\text{ D}3,\text{ and D}4/\text{the number of panicles in the main season} \end{array}$$

The grain yield and it’s component of main and ratoon crops in 2018 were already presented in the previous publications (He et al., 2019). And the relevant data in 2019 were added in **Supplemental Tables 2**, **3**.

#### 2.3.4 Extraction and determination of endogenous hormones in stems and buds

On the 15th days after leaf-cutting and spikelet-thinning treatment in 2018 and 2019, 5-6 hills with the same flowering time and size were selected from each plot. Two main tillers were selected from each hill, and approximately 2cm of stem and regenerated buds on D2, D3, and D4 of each tiller were collected in three 10 cm centrifuge tubes after removing the leaf sheath. Samples were then kept in a refrigerator at -80°C until testing.

Fresh frozen samples of stems and regenerated buds were ground into powder by adding liquid nitrogen in an ice bath, and approximately 0.05-0.1 g fresh powder sample was weighed. Then, the mixture was placed into a 2 cm centrifuge tube, and 1 ml of 80% methanol extract was added. For 15 seconds were shaken and mediated under ice bath conditions to mix evenly. A 2 ml centrifuge tube was placed in an ultrasonic cleaning instrument for ultrasonic extraction for 30 min under ice bath conditions and then centrifuged for 15 min under a centrifugal force of 12,000 rhm at 4°C. The centrifuged supernatant was removed with a 2 ml syringe, passed through a 0.22 µm filter membrane (Nylon66, Jinteng) at a uniform speed, and then the filtrate was passed stored in a 2 ml brown sampling bottle for testing. The extracted hormone samples were stored in a -80 °C ultralow temperature refrigerator. The hormonal content was detected by LCMS-8050 liquid chromatography-mass spectrometry (LCMS-8050, Shimadzu). The method of determination of plant endogenous hormones was slightly modified according to Guérard et al. (2017). The plant hormones mainly include tZ9G (trans-zeatin-9-glucoside), tZR (trans-zeatin-riboside), tZ (trans-zeatin), iP9G (N6-isopentenyl adenine-9-glucoside), iPR (Isopentenyladenine riboside), iP (N6-isopentenyl adenine), IAA (indole-3-acetic acid), GA_1_ (gibberellic acid 1), GA_3_ (gibberellic acid 1) and ABA (abscisic acid). The content of CTK was the sum of the content of tZ9G, tZR, tZ, iP9G, iPR, and iP (**Supplemental Tables 4**, **5**). The contents of these hormones were determined by the external standard method, and the recovery rates of tZ9G, tZR, tZ, iP9G, iP, iPR, GA_1_, GA_3_, IAA, and ABA were 102.2%, 88.7%, 76.5%, 86.7%, 42.9%, 71.2%, 93.0%, 136.1%, 97.1%, and 92.6%, respectively.


(2) $$\begin{array}{l} \text{CTK}\;({\text{ng g}}^{-1})=\text{tZ}9\text{G}\;({\text{ng g}}^{-1})+\text{tZR}\;({\text{ng g}}^{-1})+\text{tZ }\left({\text{ng g}}^{-1}\right)\\ +\text{iP}9\text{G}+\text{iPR}\;({\text{ng g}}^{-1})+\text{iP}\;({\text{ng g}}^{-1}) \end{array}$$

#### 2.3.5 Data analysis

Data were analyzed through analysis of variance (ANOVA) using Statistix 9.0 software. The differences between treatments or multiple comparisons were compared using the least significance difference (LSD) test at 0.05 probability levels. Sigmaplot 12.5 was used for the graphical presentation of the data.

## 3 Results

### 3.1 Weather conditions

**Figure 2:**
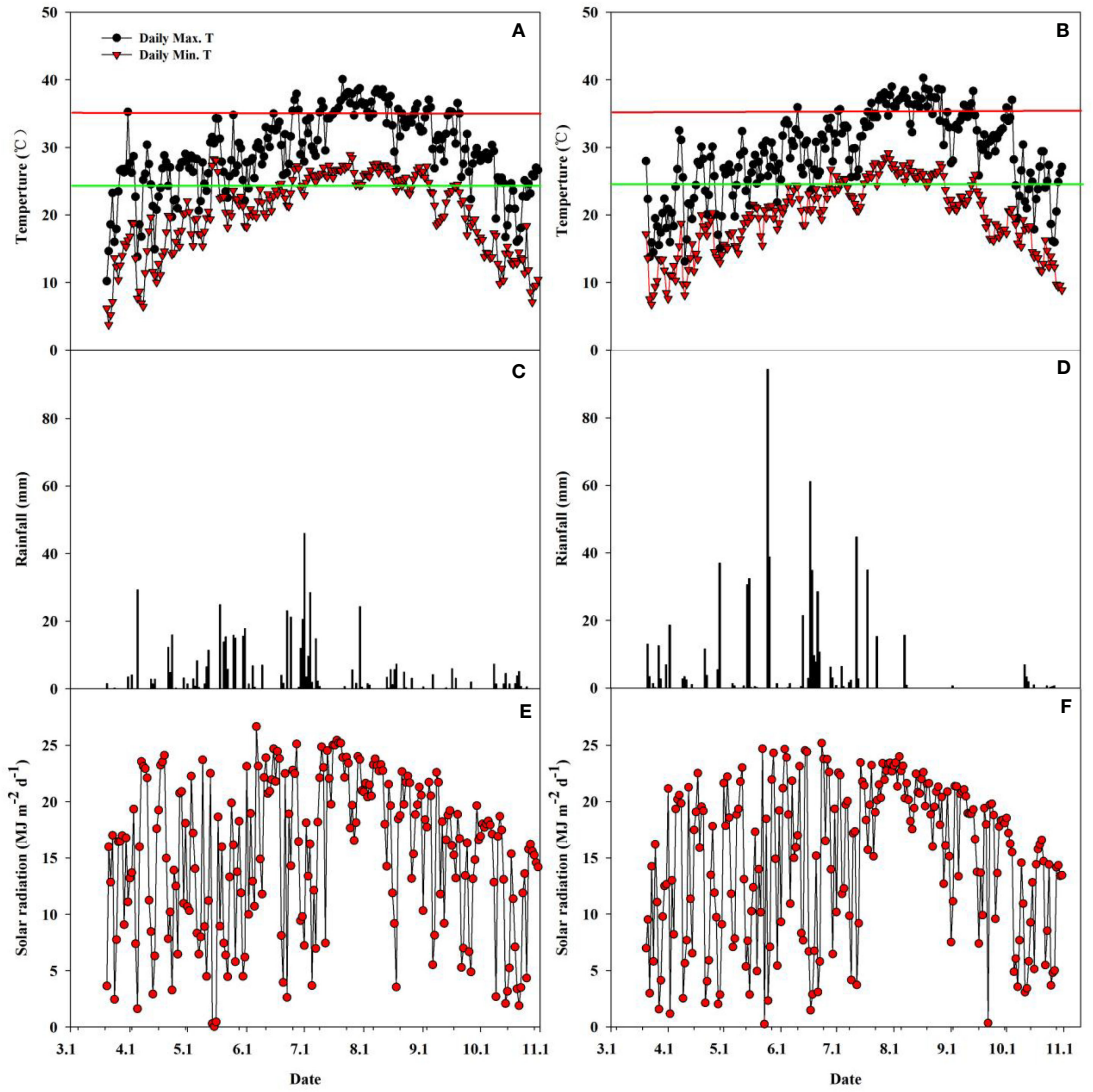
Daily minimum and maximum temperature **(A, B)**, rainfall **(C, D)**, and solar radiation **(E, F)** during the rice-growing season from seeding to maturity of ratoon crop at Qichun County, Hubei Province, China in 2018 **(A, C, E)** and 2019 **(B, D, F)**. Red horizontal lines in **Figures 1A, B** represent a critical temperature of 35°C; Green horizontal lines in **Figures 1A, B** represent the average temperature during the whole growth duration.

The rainfall, solar radiation, and daily maximum and minimum temperatures during the rice growing seasons in both years 2018 and 2019 are shown in **Figure 2**. There were evident differences in rainfall, temperature, and solar radiation between the two years during the whole main and ratoon rice growing season. The solar radiation in 2019 was 3365.7 MJ m^-2^, which was 3.2% lower than that in 2018 (3474.8 MJ m^-2^) (**Figures 2E, F**). In contrast, the total rainfall was 657.0 mm in 2019, which was 18.0% higher than that in 2018 (556.9 mm) (**Figures 2C, D**). The average daily maximum temperature, minimum temperature, and average temperature were 29.26°C, 20.20°C, and 24.15°C and 29.04°C, 19.74°C, and 23.80°C, respectively, in 2018 and 2019 (**Figures 2A, B**). Additionally, extremely high temperatures (daily maximum temperature ≥35°C) occurred more frequently in 2018 than in 2019.

### 3.2 Effects of leaf-cutting and spikelet-thinning treatments on grain yield of main season, ratoon season and annual yield, and the yield contribution of separate node to total grain yield in ratoon crop

**Figure 3:**
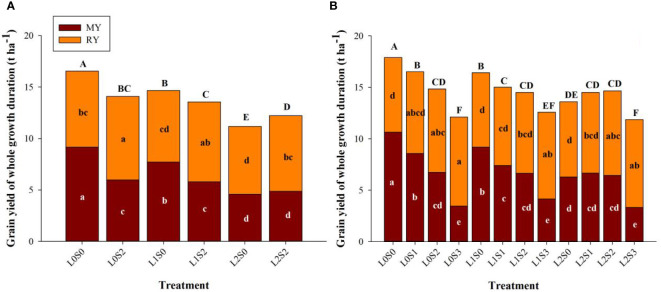
Effects of leaf cutting and spikelets thinning on grain yield in main crop, ratoon crop and annual yield in 2018 **(A)** and 2019 **(B)**. L0 represent the treatment of no leaf-cutting; L1 represent the treatment of cutting top of one leaf; L2 represent the treatment of cutting top of three leaves; S0, S1, S2 and S3 represent the treatment of thinning 0, 1/4, 1/2 and 3/4 spikelets, respectively. MY and RY represent the grain yield in main crop and ratoon crop, respectively. The different lower-case letters on the same color column and upper-case letters between different treatments in the same year all showed significant differences at the 0.05 level.

Leaf-cutting and spikelets-thinning treatments significantly affected grain yield of the main crop, ratoon crop, and annual yield (**Figure 3**). Both leaf-cutting and spikelets-thinning treatments reduced the grain yield of the main season and annual yield, the same trends were observed in 2018 and 2019. Leaf-cutting treatments decreased the grain yield of the main season, ratoon season and the annual grain yield, therein the annual yield was reduced by 19.5%. On the contrary, spikelets-thinning treatments significantly increased the grain yield in ratoon season, and the yield contribution of the ratoon season to total grain yield was averagely increased by 10.3%.

**Figure 4:**
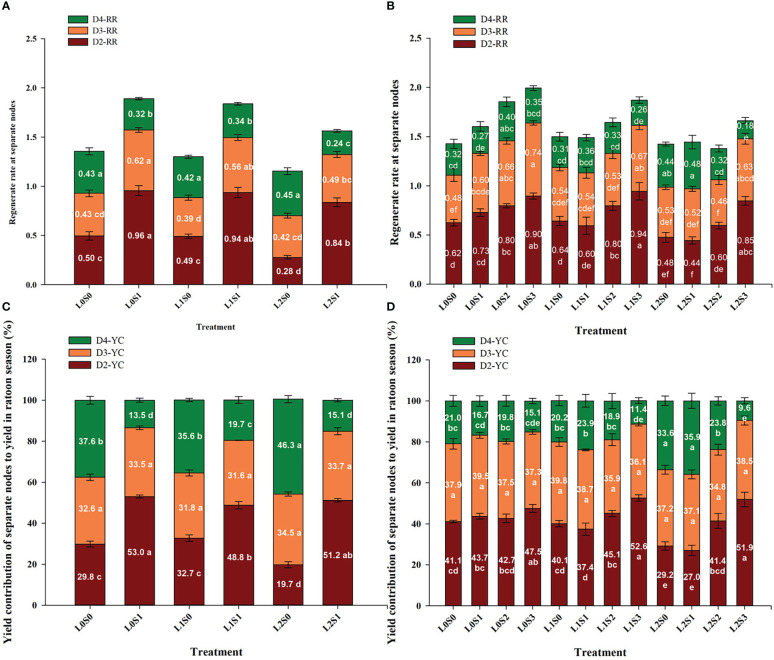
Effects of leaf cutting and spikelets thinning on regeneration rate **(A, B)** at separate nodes and yield contributions of separate node to total grain yield in ratoon crop **(C, D)** in 2018 **(A, C)** and 2019 **(B, D)**. L0 represent the treatment of no leaf-cutting; L1 represent the treatment of cutting top of one leaf; L2 represent the treatment of cutting top of three leaves; S0, S1, S2 and S3 represent the treatment of thinning 0, 1/4, 1/2 and 3/4 spikelets, respectively. D2, D3 and D4 represent the second, third and lower than third nodes from the top, respectively. D2-YC, D3-YC and D4-YC represent the yield contribution of D2, D3 and D4 node to total grain yield in ratoon crop, respectively; D2-RR, D3-RR and D4-RR represent the regeneration rate in D2, D3 and D4 nodes, respectively. The value in each stacking bar represents the mean value of yield contribution or regeneration rate at separate nodes. The different lower-case letters on the same color column showed significant differences at the 0.05 level.

The yield contribution of the separate node to the total yield in the ratoon crop was in the sequence of D2 > D3 > D4, and the same trends were observed in both years (**Figures 4C**, **3D**). The yield at D2 and D3 accounted for approximately 72.0-79.2% of the total yield, and D4 accounted for 20.8-28%. In addition, leaf-cutting and spikelet-thinning treatment significantly affected the yield contribution of D2 and D4 to total grain yield in ratoon crop, while no effect on the yield contribution to total yield was observed in D3. Furthermore, compared with no spikelet-thinning control, spikelet-thinning treatment increased the yield contribution of D2 by 23.6% and 6.4%, while decreasing the yield contribution of D4 by 23.7% and 5.5% in 2018 and 2019, respectively. In contrast, compared with no leaf-cutting control, leaf-cutting treatment decreased the yield contribution of D2 by 3.3% and 3.2%, while increasing the yield contribution of D4 by 3.7% and 4.0% in 2018 and 2019, respectively.

### 3.3 Effects of leaf-cutting and spikelet thinning on the growth of regenerated buds and regeneration rate at separate nodes

Leaf-cutting and spikelet thinning treatments significantly affected single bud length and number of buds per area at separate nodes, while there was no obvious interaction between leaf-cutting and spikelet thinning treatment (**Table 1**; **Supplemental Table 6**). In general, the spikelet-thinning treatment significantly increased the average single bud length and the number of buds per unit area. In contrast, the leaf-cutting treatment significantly decreased the average single bud length and the number of buds per unit area, and the trend was the same within two years. In addition, the same results were also observed in D2, D3, and D4. In addition, the difference in single bud length and bud number of regenerated buds between two years was mainly due to the strong rainfall and the high field water level in 2019, promoting regenerated bud growth.

**Table 1:** Effects of leaf cutting and spikelet thinning on different nodes single bud length and number of bud per area at the maturity stage of the main crop.

Year	Treatment	Leaf-cutting treatment	Spikelet-thinning treatment	D2		D3		D4	
				SBL (cm)	BN (no m^2^)	SBL (cm)	BN (no m^2^)	SBL (cm)	BN (no m^2^)
2018	L0S0	L0	S0	1.16 c	47 bc	0.89 c	44 c	0.86 c	56 cd
	L0S2	L0	S2	30.28 a	266 a	30.63 a	248 a	11.78 a	229 a
	L1S0	L1	S0	0.65 c	16 c	1.01 c	28 c	0.75 c	47 cd
	L1S2	L1	S2	16.10 b	229 a	11.68 b	191 a	4.59 b	166 ab
	L2S0	L2	S0	0.31 c	13 c	0.49 c	13 c	0.50 c	19 d
	L2S2	L2	S2	3.36 c	113 b	3.85 c	113 b	1.85 bc	110 bc
			Mean	8.64 B	113 B	8.093 B	106 B	3.405 B	104 B
2019	L0S0	L0	S0	7.81 ef	238 cde	5.35 fg	196 cd	2.93 gh	152 gh
	L0S1	L0	S1	13.75 bcd	254 bcde	15.02 bcd	238 c	5.08 ef	186 f
	L0S2	L0	S2	14.46 abc	323 ab	19.32 b	301 ab	8.79 c	290 d
	L0S3	L0	S3	19.28 a	357 a	26.42 a	348 a	12.92 a	419 a
	L1S0	L1	S0	6.43 f	192 ef	8.58 efg	194 cd	2.79 gh	118 ij
	L1S1	L1	S1	13.17 bcd	222 cdef	13.77 cde	245 bc	4.56 f	161 g
	L1S2	L1	S2	12.29 cde	270 bcd	15.29 bcd	229 cd	6.51 d	247 e
	L1S3	L1	S3	17.77 ab	295 abc	28.75 a	297 ab	10.92 b	381 b
	L2S0	L2	S0	5.02 f	117 g	3.82 g	96 e	2.24 h	97 j
	L2S1	L2	S1	5.33 f	151 fg	10.33 def	171 d	3.40 g	128 hi
	L2S2	L2	S2	9.21 def	213 def	14.32 bcd	230 c	6.14 de	146 gh
	L2S3	L2	S3	15.58 abc	317 ab	16.55 bc	309 a	9.46 c	332 c
			Mean	11.677 A	246 A	14.793 A	238 A	6.310 A	221 A

L0, represents the treatment of no leaf-cutting; L1, represents the treatment of cutting the first leaf from the top; L2, represent the treatment of cutting top three leaves; S0, S1, S2 and S3 represent the treatment of thinning 0, 1/4, 1/2 and 3/4 spikelets, respectively. SBL, represent single bud length; BN, represent number of bud per area. D2, D3 and D4 represent the second, third and lower than third nodes from the top, respectively. Data followed by different lower-case letters at different treatment in the same year and different upper-case letters at the same column denote significant differences between treatments at the 5% level according to LSD test.

In general, the spikelet-thinning treatment significantly increased the regeneration rate; in contrast, the leaf-cutting treatment evidently decreased the regeneration rate (**Figure 4**). Compared with the no spikelet-thinning treatment, the regeneration rate under the spikelet-thinning treatment was increased by 35.9% and 14.5% in 2018 and 2019, respectively. The regeneration rate under the leaf-cutting treatment decreased by 13.0% and 8.9% in 2018 and 2019, respectively; compared to that under the no leaf-cutting treatment. Compared with the no spikelet-thinning treatment, the regeneration rate in D2 and D3 under the spikelet-thinning treatment was increased by 128.0% and 33.2% and 27.6% and 16.7% in 2018 and 2019, respectively. The regeneration rate in D4 under the spikelet-thinning treatment decreased by 43.6% and 6.3% in 2018 and 2019, respectively. In addition, the leaf-cutting treatment significantly decreased the regeneration rate of D2 and D3 compared to the no leaf-cutting treatment. However, there was no significant difference in the D4 regeneration rate between the different leaf-cutting treatments.

### 3.4 Changes in endogenous hormone contents of stems and buds in separate nodes and their relationship with the growth of regenerated buds at 15 days after leaf cutting and spikelet thinning treatment

Spikelet-thinning treatment significantly increased the CTK content of stems and buds in D2, D3, and D4, and the GA_3_ and IAA content of stems and buds in D3, on the contrary, significantly decreased the GA_1_ content in D3 and ABA content in D4 (**Tables 2**, **3**). In addition, the effects of spikelet-thinning on the GA_1_ and GA_3_ contents in stems and buds in D2, the GA_1_ content in D3, and the IAA content in D4 were inconsistent between the two years. Leaf-cutting treatment significantly reduced the CTK content in D2, D3, and D4 of stems and buds by 39.0%, 27.1%, 26.8%, and 42.7%, 23.1% and 3.8% in 2018 and 2019, respectively. Moreover, there was an obvious interaction between leaf cutting, spikelet thinning, and node position on the content of endogenous hormones.

**Table 2:** Changes of endogenous hormone content in stems and buds at separate nodes at 15 days after leaf-cutting and spikelet-thinning treatment in 2018.

Node	Treatment	Leaf-cutting treatment	Spikelet-thinning treatment	GA_1_ (ng g^-1^)	GA_3_ (ng g^-1^)	IAA (ng g^-1^)	ABA (ng g^-1^)	CTK (ng g^-1^)
D2	L0S0	L0	S0	11.332 c	0.519 c	9.094 c	2.883 b	40.891 b
	L0S2	L0	S2	18.020 b	0.661 b	24.837 a	2.320 de	69.903 a
	L1S0	L1	S0	9.613 c	0.438 c	13.185 b	3.350 a	28.874 c
	L1S2	L1	S2	21.448 a	0.524 c	14.524 b	2.710 bc	43.631 b
	L2S0	L2	S0	15.711 b	0.677 b	13.188 b	2.352 cd	20.702 d
	L2S2	L2	S2	17.546 b	0.826 a	15.704 b	1.967 e	42.157 b
	**Mean**			**15.612 B**	**0.608 B**	**15.089 C**	**2.597 B**	**41.026 C**
D3	L0S0	L0	S0	22.250 a	0.410 bc	10.646 d	2.296 c	75.369 a
	L0S2	L0	S2	10.109 d	1.006 a	16.355 c	9.365 a	75.307 a
	L1S0	L1	S0	17.589 b	0.552 b	19.397 b	1.529 d	57.710 c
	L1S2	L1	S2	12.400 c	0.540 b	21.392 b	2.966 b	63.434 b
	L2S0	L2	S0	18.507 b	0.328 c	15.470 c	2.048 c	46.635 e
	L2S2	L2	S2	12.558 c	0.552 b	24.117 a	1.582 d	52.083 d
	**Mean**			**15.569 B**	**0.565 B**	**17.896 B**	**3.298 A**	**61.756 B**
D4	L0S0	L0	S0	15.575 f	0.409 b	16.489 c	2.385 a	76.084 b
	L0S2	L0	S2	18.723 e	0.496 b	24.624 b	1.648 c	81.383 a
	L1S0	L1	S0	22.435 b	0.597 b	20.189 bc	2.448 a	59.999 d
	L1S2	L1	S2	21.061 c	0.346 b	19.334 c	2.026 b	71.184 c
	L2S0	L2	S0	19.664 d	0.312 b	34.349 a	1.404 d	45.545 f
	L2S2	L2	S2	24.119 a	2.554 a	17.004 c	1.585 cd	54.186 e
	**Mean**			**20.263 A**	**0.786 A**	**21.998 A**	**1.916 C**	**64.730 A**
	ANOVA		N	*	**	***	***	*
			L	***	*	***	**	***
			S	***	***	***	***	***
			L*N	***	**	***	***	***
			S*N	***	***	***	ns	***
			L*S	***	***	***	***	***
			L*S*N	***	***	***	***	***

L0, represent the treatment of no leaf-cutting; L1, represents the treatment of cutting the first leaf from the top; L2, represent the treatment of cutting top three leaves; S0 and S2 represent the treatment of thinning 0 and 1/2 spikelets, respectively. D2, D3 and D4 represent the second, third and lower than third nodes from the top, respectively. The content of CTK was sum of the content of TZ9G, TZ, TZR, IP9G, IPR and IP. Data followed by different lower-case letters in the same node and different upper-case letters within one column denote significant differences between treatments at the 5% level according to LSD test. ***, ** and * represents the significant difference at the 0.1%, 1% and 5% level according to LSD test, respectively. The bold values represent the mean value of all treatment at separate nodes.

**Table 3:** Changes of endogenous hormone content in stems and buds at separate nodes at 15 days after leaf-cutting and spikelet-thinning treatment in 2019.

Node	Treatment	Leaf cutting treatment	Spikelet- thinning treatment	GA_1_ (ng g^-1^)	GA_3_ (ng g^-1^)	IAA (ng g^-1^)	ABA(ng g^-1^)	CTK (ng g^-1^)
D2	L0S0	L0	S0	14.053 d	0.534 ab	13.67 d	3.018 b	29.03 f
	L0S1	L0	S1	15.745 cd	0.637 ab	13.99 d	2.996 bc	58.25 d
	L0S2	L0	S2	10.942 e	0.757 a	14.58 d	2.781 bc	67.42 c
	L0S3	L0	S3	14.256 cd	0.383 b	33.82 c	2.534 c	115.68 a
	L1S0	L1	S0	5.103 fg	0.610 ab	88.18 b	0.929 f	19.07 g
	L1S1	L1	S1	5.086 fg	0.503 ab	80.09 b	1.143 ef	30.55 f
	L1S2	L1	S2	19.272 b	0.575 ab	17.80 d	4.233 a	62.57 cd
	L1S3	L1	S3	22.076 a	0.368 b	22.10 d	2.962 bc	92.59 b
	L2S0	L2	S0	6.566 f	0.612 ab	101.49 a	1.247 ef	9.59 h
	L2S1	L2	S1	4.731 g	0.336 b	86.45 b	1.536 de	10.55 h
	L2S2	L2	S2	15.504 cd	0.532 ab	17.20 d	1.734 d	40.13 e
	L2S3	L2	S3	15.952 c	0.441 b	11.98 d	1.985 d	65.43 cd
	**Mean**			**12.440 A**	**0.524 A**	**41.779 A**	**2.258 A**	**50.070 C**
D3	L0S0	L0	S0	7.128 e	0.226 e	11.553 g	2.281 bc	67.670 de
	L0S1	L0	S1	8.770 de	0.319 de	11.351 g	1.930 cd	77.299 bcd
	L0S2	L0	S2	9.072 cde	0.294 de	11.459 g	2.234 bc	77.145 bcd
	L0S3	L0	S3	10.007 cd	0.352 de	20.967 bc	1.933 cd	96.162 a
	L1S0	L1	S0	9.971 cd	0.250 e	17.641 def	3.005 a	71.090 cde
	L1S1	L1	S1	10.353 cd	0.254 e	19.303 cde	2.388 b	73.566 cd
	L1S2	L1	S2	10.988 bc	1.188 b	20.436 cd	2.311 bc	80.441 bc
	L1S3	L1	S3	12.654 ab	1.387 a	24.438 a	1.772 de	87.315 ab
	L2S0	L2	S0	12.488 ab	0.354de	15.563 f	1.678 def	35.018 f
	L2S1	L2	S1	13.213 a	0.420 d	16.099 f	1.339 fg	38.606 f
	L2S2	L2	S2	13.844 a	0.576 c	17.166 ef	1.225 g	40.850 f
	L2S3	L2	S3	10.214 cd	0.655 c	23.714 ab	1.405 efg	62.501 e
	**Mean**			**10.725 C**	**0.523 A**	**17.474 B**	**1.959 B**	**67.305 B**
D4	L0S0	L0	S0	16.044 a	0.455 bc	17.419 bcde	2.416 b	82.39 gh
	L0S1	L0	S1	11.927 b	0.317 de	18.693 abcd	1.858 cde	94.56 def
	L0S2	L0	S2	11.117 bc	0.220 ef	19.000 abc	1.827 cde	104.38 cd
	L0S3	L0	S3	12.736 b	0.449 bcd	22.252 a	1.255 g	114.12 a
	L1S0	L1	S0	11.050 bc	0.318 de	12.892 f	2.220 bc	76.06 h
	L1S1	L1	S1	9.658 bc	0.349 cde	13.186 f	1.950 cde	87.03 fg
	L1S2	L1	S2	11.692 bc	0.491 ab	12.446 f	1.621 efg	110.35 bc
	L1S3	L1	S3	8.722 c	0.299 e	19.953 ab	1.365 fg	120.74 a
	L2S0	L2	S0	10.924 bc	0.301 e	14.394 ef	2.963 a	75.17 h
	L2S1	L2	S1	11.638 bc	0.601 a	14.557 ef	2.108 bcd	92.06 efg
	L2S2	L2	S2	11.218 bc	0.472 abc	14.675 def	1.813 cde	98.99 de
	L2S3	L2	S3	10.307 bc	0.160 f	15.478 cdef	1.752 def	102.98 cd
	**Mean**			**11.419 B**	**0.369 B**	**16.245 B**	**1.29 B**	**95.736 A**
ANOVA			N	***	ns	***	**	***
			L	ns	***	***	***	***
			S	***	***	***	***	***
			L*N	***	***	***	***	***
			S*N	***	***	***	***	***
			L*S	***	***	***	***	***
			L*S*N	***	***	***	***	**

L0, represent the treatment of no leaf-cutting; L1, represents the treatment of cutting the first leaf from the top; L2, represent the treatment of cutting top three leaves; S0, S1, S2 and S3 represent the treatment of thinning 0, 1/4, 1/2 and 3/4 spikelets, respectively. The content of CTK was sum of the content of TZ9G, TZ, TZR, IP9G, IPR and IP. D2, D3 and D4 represent the second, third and lower than third nodes from the top, respectively. Data followed by different lower-case letters in the same node and different upper-case letters within one column denote significant differences between treatments at the 5% level according to LSD test. *** and ** represents the significant difference at the 0.1% and 1% level according to LSD test, respectively, ns represents no significant difference. The bold values represent the mean value of all treatment at separate nodes.

The changes in endogenous hormone content in different nodes were closely related to the sprouting of regenerated buds (single bud length and the number of buds per area) (**Table 4**). The CTK contents of stems and buds in D2, D3, and D4 were positively correlated with the length of a single bud and the number of regenerated buds per unit area at each node at the maturity stage of the main crop, and the trend was the same within two years. Furthermore, the GA_1_ content in stems and buds in D2 was significantly positively correlated with single bud length and the number of regenerated buds per area in both years. However, the relationships between the GA_1_, GA_3_ and IAA contents in stems and buds on D3 and their single bud length and bud number and the IAA and ABA contents of stems and buds on D4 and their single bud length and bud number were inconsistent between the two years.

**Table 4:** Correlation between endogenous hormones of stems and buds at each node 15 days after treatment and single bud length and bud number per area in maturity stage of the main crop in 2018 and 2019.

Year	Node	r	GA_1_ (ng g^-1^)	GA_3_ (ng g^-1^)	IAA (ng g^-1^)	ABA (ng g^-1^)	CTK (ng g^-1^)
2018	D2	SBL (cm)	0.532**	0.073ns	0.704***	-0.226ns	0.809***
		BN (no m^-2^)	0.727***	0.162ns	0.699***	-0.331ns	0.843***
	D3	SBL (cm)	-0.717***	0.816***	0.036ns	0.941***	0.546**
		BN (no m^-2^)	-0.826***	0.732***	0.295ns	0.778***	0.500*
	D4	SBL (cm)	-0.155ns	-0.148ns	0.064ns	-0.281ns	0.657***
		BN (no m^-2^)	0.021ns	0.049ns	-0.172ns	-0.211ns	0.667***
2019	D2	SBL (cm)	0.581***	-0.084ns	-0.565***	0.417*	0.910***
		BN (no m^-2^)	0.604***	-0.127ns	-0.718***	0.531***	0.860***
	D3	SBL (cm)	0.110ns	0.532***	0.530***	-0.126ns	0.656***
		BN (no m^-2^)	-0.210ns	0.258ns	0.386*	-0.025ns	0.710***
	D4	SBL (cm)	-0.160ns	-0.031ns	0.605***	-0.657***	0.785***
		BN (no m^-2^)	-0.102ns	-0.039ns	0.604***	-0.710***	0.777***

SBL, represent the single bud length; BN, represent the number of buds. D2, D3 and D4 represent the second, third and lower than third nodes from the top, respectively. The content of CTK was sum of the content of TZ9G, TZ, TZR, IP9G, IPR and IP. *** represents the significant difference at the 0.1% level according to LSD test, ** represents the significant difference at the 1% level according to LSD test, * represents the significant difference at the 5% level according to LSD test, ns represents no significant difference.

## 4 Discussion

Our previous study indicated that leaf-cutting and spikelet-thinning treatments significantly affected the grain yield in ratoon crops, mainly through affecting the number of panicles per unit area or regeneration rate (He et al., 2019). This study showed that leaf-cutting and spikelet-thinning treatments affected the regeneration rate at separate nodes (**Figures 4A, B**). And there was a significantly positive correlation between the regeneration ability of each node and the grain yield of ratoon crops at separate nodes (data not shown). Therefore, leaf-cutting and spikelet-thinning treatments affected the grain yield in ratoon crop *via* regulating the regeneration rate at the separate node.

**Figure 5:**
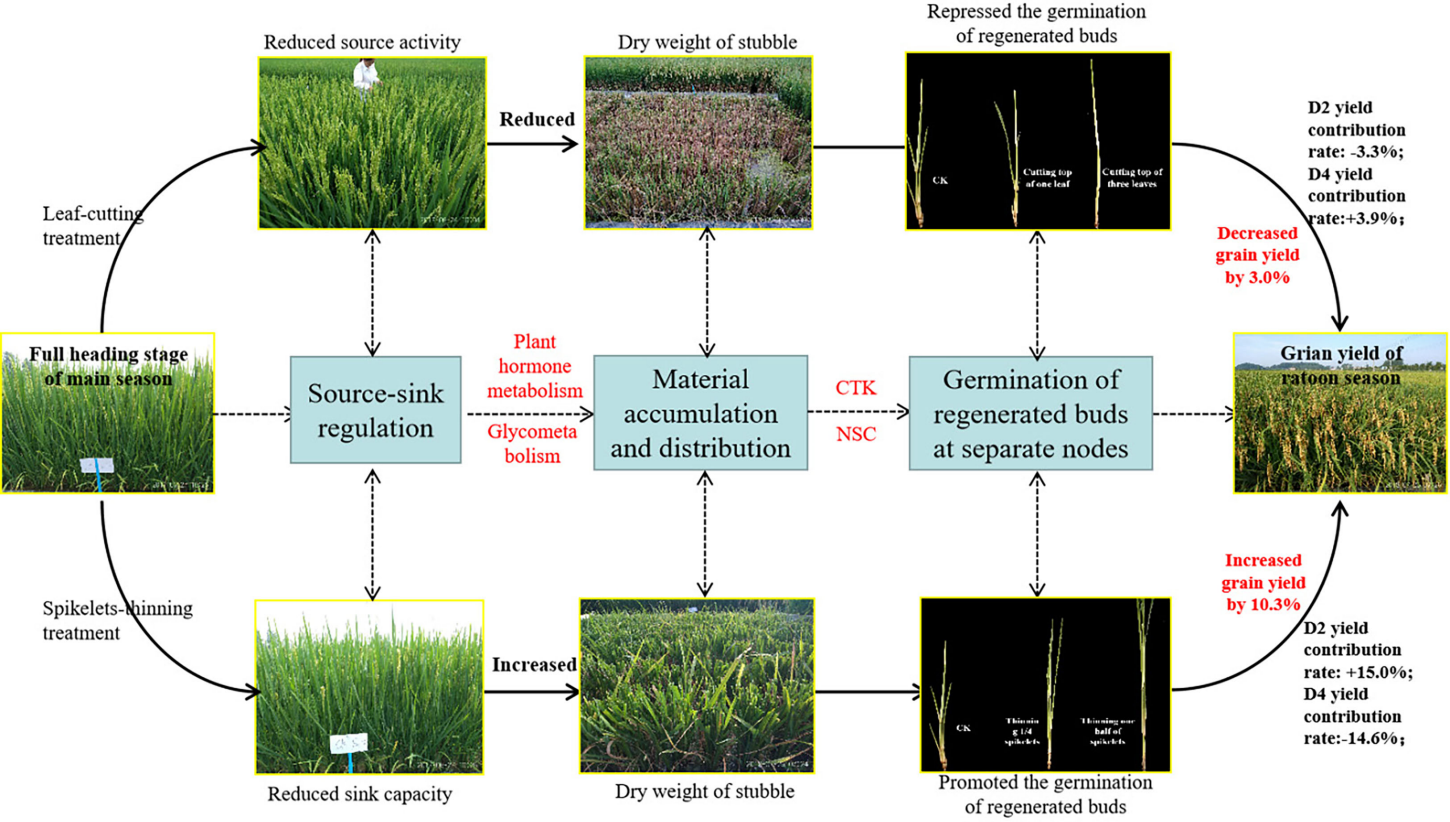
Pathway diagram for the effect of leaf-cutting treatment and spikelets-thinning treatment on the grain yield in ratoon season.

The contribution of separate node yields to the total yield of the ratoon crop, the overall performance was in the sequence of D2>D3>D4, and the contribution of D2 and D3 made up approximately 80% of the total yield in the ratoon crop (**Figures 4C, D**). Similar results were observed by Dong et al. (2017) and Xu et al. (2020). Furthermore, the effects of leaf-cutting and spikelet-thinning treatments on the grain yield in ratoon crops were mainly by changing the yield contribution of D2 and D4 to the total grain yield in ratoon crop (**Figures 4C, D**). And it was reported that the dry weight and NSC content of stubble at the maturity stage of the main crop were significantly positively correlated with the regeneration rate and the yield of the ratoon crop (Chen et al., 2018; He et al., 2019; **Figure 5**). Additionally, after heading of the main crop, the differentiation and growth dynamics of axillary buds at separate nodes occurred from top to bottom, and the regenerated buds on the top (D2 and D3) developed much faster than the other buds (D4) (Xu et al., 2020). It was consistent with our result showing that the single bud length and number of bud per area were performed in the sequence of D2>D3>D4 (**Table 1**). It was estimated that 60.7-82.5% of the photo-assimilate stored in stubble was used for the sprouting and growth of regenerated buds, especially the regenerated buds at the top nodes (D2 and D3) (Liu and Wang, 1993; Wang, 2011). Therefore, the effect of leaf-cutting and spikelet-thinning treatments on the yield contribution of separate nodes to total yield in ratoon crop might be closely related to the amount of photo-assimilate residues in stubble at each node during the harvest of the main crop. However, the contribution of photo-assimilate stored in stubble to the yield of ratoon crop at separate nodes is rarely reported, which needs to be further explored. Additionally, the contribution rate of D4 node yield to the total yield of ratoon crop under control treatment (L0S0) in 2018 was 37%, significantly higher than that in 2019 (21%) (**Figure 4**). Some studies have shown that failure to maintain sufficient water supply in the field after the harvest of the main crop would lead to slow growth of regenerated buds and even death caused by water loss, which resulted in a significant reduction in the yield of ratoon crop (Xiong and Chinawong, 1995; Setiawan et al., 2014). Moreover, the rainfall in 2018 was significantly lower than that in 2019, especially after the harvest of the main crop (**Figure 2**). Therefore, the difference in the contribution of different node yields to the total yield in the ratoon season between the two years under L0S0 may be closely related to the rainfall after the harvest of the main crop.

It is well known that the yield of the ratoon crop depends on the number of effective panicles at each node to a certain extent, and the number of regenerated panicles is limited by the number of regenerated buds germinating. It is widely known that the sprouting of buds is mainly induced and regulated by endogenous hormone signals (Chatfield et al., 2000; Xu et al., 2020). Our study indicated that the spikelet-thinning treatment significantly increased the CTK content of stems and buds and the length and number of regenerated buds at each node, while the leaf-cutting treatment significantly decreased the CTK content of stems and buds and the length and number of regenerated buds at each node (**Tables 2**, **3**). The content of CTK was positively correlated with the length and number of regenerated buds at each node (**Table 4**). This result was consistent with Liu et al. (2011), who showed that IAA and CTK (Z+ZR) were the two main signals regulating the sprouting and dormancy of regenerated buds, not ABA. Furthermore, it was reported that CTK might act independently to regulate bud growth under cytokinin signaling mutants rather than as a second messenger for auxin (Chatfield et al., 2000). The results showed that the contents of GA_1/3_ and iPAs in each node of the main stem at the maturity stage of the main crop had a significant effect on the regeneration rate and showed a very significant hyperbolic regression correlation, in which the correlation of GA_1/3_ was better than that of iPAs (Cheng et al., 2003). In addition, it was demonstrated that tiller bud growth was not regulated by CTK derived from roots; in contrast, CTKs were biosynthesized mainly in the nodes and subsequently delivered to tiller buds (Liu et al., 2011). Thus, the changes in CTK content in stems and buds caused by leaf cutting and spikelet thinning were the main signals affecting the sprouting difference of regenerated buds (**Figure 5**).

In general, the triggers outbreak of the regenerated bud could be observed after the harvest of the main crop (Vergara, 1988; Wang et al., 2020). In this study, we tested phytohormones concentrations at 15 days after the heading of the main crop (**Tables 2**, **3**). It was reported that panicle differentiation of the regenerated buds starts at 15 days after the heading stage of the main crop (Lin et al., 2015; Xu et al., 2020). In addition, our previous study showed that there were significant differences in bud length and bud number at each node among treatments at 16 days after treatment in 2017 and 2018 (He et al., 2019). Thus, we measured the content of endogenous hormones in stem buds at each node at 15 days after treatment. Besides, in the actual production of ratoon rice, it is impossible for us to promote the occurrence of regenerated buds by leaf or spikelets removal. This study indicated that endogenous hormones regulate the sprouting of regenerated buds at separate nodes after leaf cutting or spikelet thinning treatments (**Tables 2**, **3**, 4). Furthermore, many researches exhibited that exogenous application the plant growth regulators (GA_3_, IAA and humic acid, etc.) could regulate the regeneration ability and yield of ratoon crop (Rounds et al., 2007; Xie et al., 2017). Therefore, inspired by the change in endogenous hormone content, plant growth regulators might be used to regulate the occurrence of regenerated buds in the actual production of ratoon rice.

Bud outgrowth occurs concomitantly with starch-reserve mobilization in stem tissues, high activity of sugar-metabolizing enzymes, and increased sugar absorption. Since CTK responses are associated with active growth or the activation of biological processes, a possible link to the carbohydrate supply has been suggested. It was demonstrated that CTK synergistically induces the production of extracellular invertase and hexose transporters, which are functionally coupled to provide carbohydrates to sink tissues and are involved in phloem unloading (Roitsch and Ehne, 2000). It was reported that sugar-induced lateral bud outgrowth was in part, promoted by the induction of CTK-mediated vacuolar invertase activity (Salam et al., 2021). Previous research showed significant differences in stubble dry weight and NSC content at the maturity stage of the main crop under the different leaf-cutting and spikelet-thinning treatments (He et al., 2019). Therefore, the effect of leaf-cutting and spikelet thinning on the sprouting of regenerated buds may be realized by regulating the transport and distribution of assimilates in the main season induced by CTK (**Figure 5**). However, the relationship between CTK and assimilate transport at each node has not been reported.

In general, more than one hormone is implicated in the sprouting of buds, and thus, coordination of their overlapping activities is crucial (Azizi et al., 2015; Zha et al., 2019; Xu et al., 2020). Interestingly, our study showed that leaf-cutting and spikelet thinning had significant effects on GAs, IAA, ABA and CTK in stems and buds at each node; among them, the sprouting of regenerated buds at node D2 was mainly affected by the content of CTK, while D3 was regulated primarily by GAs and CTK, and D4 was mainly regulated by ABA and CTK (**Tables 2**, **3**). It was documented that the application of bud-promoting fertilizer promoted the sprouting of regenerated buds, which may be related to increasing the content of GA_3_ in stems and buds, reducing the content of ABA, increasing the activity of POD and the content of soluble protein in stems, then promoting the transport of nutrients to buds (Liu et al., 1998). Previous studies indicated that CTK interacts with different phytohormones, such as IAA (Li and Tang, 2003), ethylene (Simmons et al., 1992), SLs (Chatfield et al., 2000), GAs (Sae and Hitoshi, 2001), and ABA (Izhaki et al., 1996). Some research suggests that both SLs (strigolactones) and CTK can interact directly in buds to control bud outgrowth, converging at a common target in the bud (Dun et al., 2013). In addition, it was reported that the contents of ABA and ZR in stems before harvest of the main crop were positively correlated with the regeneration rate, while (IAA+GA)/ABA and (IAA+GA+ZR)/ABA were significantly negatively correlated with the regeneration rate (Xiao, 2002). Previous studies have shown that axillary bud sprouting was always accompanied by changes in the concentration of endogenous hormones, such as IAA, ABA, CTK and GAs, and these hormones reached a new balance after a certain period of sprouting (Li and Tang, 2003; Qin et al., 2009; Nie et al., 2016). Additionally, the differentiation and growth dynamics of axillary buds at different nodes occurred from the top (D2 and D3) to the bottom (D4) (Xu et al., 2020). Therefore, the difference in endogenous hormones regulating the sprouting of regenerated buds between D2, D3, and D4 may be closely related to the differences in the sprouting time of regenerated buds at separate nodes. However, the regulatory networks of endogenous hormones between CTK and IAA, ABA and GAs in the sprouting of regenerated buds at separate nodes are not clear and need to be further studied. Concurrently, it was reported that BR (brassinosteroid) might be an endogenous signal substance regulating the sprouting of regenerated buds at different nodes (Xu et al., 2020). However, the relationship between the BR content in different nodes and the sprouting of regenerated buds needs to be further addressed.

## 5 Conclusions

Our results showed that the contribution of different node yields to the total yield of the ratoon crop, was in the sequence of D2>D3>D4, and the contribution of D2 and D3 made up approximately 80% of the total yield in the ratoon crop. In addition, leaf-cutting and spikelet-thinning treatments had significant effects on the yield contribution of D2 and D4 to the total grain yield in the ratoon crop. However, no significant effect on the contribution of D3 yield to the total grain yield in the ratoon crop. This might be closely related to the amount of photo-assimilate residues in stubble at each node during the harvest of the main crop. Analysis revealed that leaf-cutting and spikelet-thinning treatments mainly changed the CTK content in stems and buds at each node, which might change the transport of assimilates in stems and buds and thus affect the sprouting of regenerated buds and grain yield of the ratoon crop at different nodes. Interestingly, our study showed that the sprouting of regenerated buds at the D2 node was mainly affected by the content of CTK, while D3 was mainly regulated by GAs and CTK, and ABA and CTK mainly regulated D4. However, the regulatory networks of endogenous hormones between CTK and IAA, ABA and GAs in the sprouting of regenerated buds at different nodes are not clear and need to be further addressed.

## Data availability statement

The original contributions presented in the study are included in the article/**Supplementary Material**. Further inquiries can be directed to the corresponding author.

## Author contributions

AH conducted the field experiments and wrote the manuscript. MJ participated data collection and data analysis. JM and SP provided advice on experimental implementation. LN conceived and supervised the field experiments and revised manuscript. All authors contributed to the article and approved the submitted version.
