# Supplementary material for: Effects of source-sink regulation and nodal position of the main crop on the sprouting of regenerated buds and grain yield of ratoon rice
Source: Front Plant Sci. 2023 Mar 27;14:1043354. doi: 10.3389/fpls.2023.1043354 (PMC10083393; doi:10.3389/fpls.2023.1043354)
Supplement: Supplementary file 1 [file Table_1.docx]

**Supplemental Table 1.** Growth stages and durations of main and ratoon crops under different leaf-cutting and spikelet-thinning treatments in 2019.

| Treatment | Leaf-  cutting | Spikelet-  thinning | Main crop season (d) | | |  | Ratoon crop season (d) | | | Whole crop duration (d) |
| --- | --- | --- | --- | --- | --- | --- | --- | --- | --- | --- |
|  |  |  | TP-MHD | MHD-MMS | Main crop duration |  | MMS-RHD | RHD-RMS | Ratoon crop duration |  |
| L0S0 | L0 | S0 | 71 | 32 | 133 |  | 23 | 45 | 68 | 201 |
| L0S1 | L0 | S1 | 71 | 28 | 129 |  | 27 | 41 | 68 | 197 |
| L0S2 | L0 | S2 | 71 | 24 | 125 |  | 26 | 42 | 68 | 193 |
| L0S3 | L0 | S3 | 71 | 22 | 123 |  | 21 | 40 | 61 | 184 |
| L1S0 | L1 | S0 | 71 | 35 | 136 |  | 30 | 35 | 65 | 201 |
| L1S1 | L1 | S1 | 71 | 29 | 130 |  | 26 | 41 | 67 | 197 |
| L1S2 | L1 | S2 | 71 | 28 | 129 |  | 24 | 40 | 64 | 193 |
| L1S3 | L1 | S3 | 71 | 22 | 123 |  | 22 | 39 | 61 | 184 |
| L2S0 | L2 | S0 | 71 | 35 | 136 |  | 38 | 43 | 81 | 217 |
| L2S1 | L2 | S1 | 71 | 35 | 136 |  | 32 | 44 | 76 | 212 |
| L2S2 | L2 | S2 | 71 | 31 | 132 |  | 22 | 43 | 65 | 197 |
| L2S3 | L2 | S3 | 71 | 24 | 125 |  | 21 | 38 | 59 | 184 |

Note：L0S0: no leaf-cutting and no spikelet-thinning; L0S1: no leaf-cutting and thinning 1/4 spikelets; L0S2: no leaf-cutting and thinning one half of spikelets; L0S3: no leaf-cutting and thinning 3/4 spikelets; L1S0: cutting the first leaf from the top and no spikelet-thinning; L1S1: cutting the first leaf from the top and thinning 1/4 spikelets; L1S2: cutting the first leaf from the top and thinning one half of spikelets; L1S3: cutting the first leaf from the top and thinning 3/4 spikelets; L2S0: cutting top three leaves and no spikelet-thinning; L2S1: cutting top three leaves and thinning 1/4 spikelets; L2S2: cutting top three leaves and thinning one half of spikelets; L2S3: cutting top three leaves and thinning3/4 spikelets. TP represent the stage of transplanting; MHD and RHD represent the stage of full heading in the main season and ratoon crop, respectively; MMS and RMS represent the satge of maturity in the main season and ratoon crop, respectively.

**Supplemental Table 2.** The grain yield and its components of main season under different leaf-cutting and spikelet-thinning treatments in 2019.

| Treatment | Leaf-  cutting | Spikelets-  thinning | Grain yield  (t ha^-1^) | Dry matter weight (t ha^-1^) | Harvest index (%) | Panicles  (No m^-2^) | Spikelet panicle^-1^ | 1000-grain weight (g) | Filled grain rate (%) |
| --- | --- | --- | --- | --- | --- | --- | --- | --- | --- |
|  |  |  |  |  |  |  |  |  |  |
| L0S0 | L0 | S0 | 10.42 a | 19.0 a | 56.0 ab | 297 ab | 154.2 a | 25.2 a | 79.3 ab |
| L0S1 | L0 | S1 | 8.73 bc | 17.0 b | 50.4 bc | 289 ab | 119.2 b | 25.7 a | 83.4 a |
| L0S2 | L0 | S2 | 6.71 de | 16.7 bc | 40.3 e | 295 ab | 98.0 c | 25.1 a | 80.5 ab |
| L0S3 | L0 | S3 | 3.18 g | 16.7 bc | 21.1 g | 314 a | 48.3 d | 23.9 bc | 81.8 a |
| L1S0 | L1 | S0 | 9.36 b | 16.1 bc | 57.0 a | 272 b | 152.2 a | 25.7 a | 74.2 bc |
| L1S1 | L1 | S1 | 8.18 c | 15.4 cd | 48.1 cd | 314 a | 104.4 bc | 25.8 a | 76.5 abc |
| L1S2 | L1 | S2 | 6.74 de | 15.4 cd | 43.0 de | 295 ab | 92.1 c | 25.4 a | 82.5 a |
| L1S3 | L1 | S3 | 4.19 f | 14.4 de | 28.5 f | 304 ab | 58.1 d | 25.1 ab | 80.5 ab |
| L2S0 | L2 | S0 | 8.16 c | 13.1 ef | 47.8 cd | 278 ab | 151.5 a | 23.7 c | 54.5 d |
| L2S1 | L2 | S1 | 7.26 d | 13.4 ef | 49.6 c | 275 b | 117.6 b | 25.2 a | 71.2 c |
| L2S2 | L2 | S2 | 6.17 e | 13.9 de | 46.1 cde | 310 ab | 92.2 c | 25.0 ab | 78.2 abc |
| L2S3 | L2 | S3 | 3.73 fg | 11.93 f | 27.9 f | 300 ab | 51.1 d | 24.7 abc | 76.0 abc |
| ANOVA | | L | ns | *** | ns | ns | ns | ns | *** |
|  |  | S | *** | ** | *** | ns | *** | * | *** |
|  |  | L*S | ** | ns | ** | ns | ns | ns | ** |

Note：L0S0: no leaf-cutting and no spikelet-thinning; L0S1: no leaf-cutting and thinning 1/4 spikelets; L0S2: no leaf-cutting and thinning one half of spikelets; L0S3: no leaf-cutting and thinning 3/4 spikelets; L1S0: cutting the first leaf from the top and no spikelet-thinning; L1S1: cutting the first leaf from the top and thinning 1/4 spikelets; L1S2: cutting the first leaf from the top and thinning one half of spikelets; L1S3: cutting the first leaf from the top and thinning 3/4 spikelets; L2S0: cutting top three leaves and no spikelet-thinning; L2S1: cutting top three leaves and thinning 1/4 spikelets; L2S2: cutting top three leaves and thinning one half of spikelets; L2S3: cutting top three leaves and thinning3/4 spikelets. Data followed by different lower-case letters denote signiﬁcant differences between treatments at the 5% level according to LSD test. ***, ** and * represents the significant difference at the 0.1%, 1% and 5% level according to LSD test, respectively. ns represents no significant difference.

**Supplemental Table 3.** Effects of leaf-cutting and spikelet-thinning on grain yield and it’s component of ratoon crop at separate nodes in 2019.

| Node | Treatment | Leaf-cutting | Spikelets-thinning | Yield  (t ha^-1^) | Biomass (t ha^-1^) | Harvest index (%) | Panicles per m^2^ | Spikelets panicles^-1^ | Filled grain rate (%) | 1000-grain weight (g) |
| --- | --- | --- | --- | --- | --- | --- | --- | --- | --- | --- |
| D2 | L0S0 | L0 | S0 | 2.99 cde | 4.44 d | 58.0 cd | 185 de | 68 c | 84.5 ab | 24.2 abc |
|  | L0S1 | L0 | S1 | 3.48 bc | 5.25 bc | 57.0 cde | 210 cd | 74 bc | 82.2 bc | 23.4 bcde |
|  | L0S2 | L0 | S2 | 3.44 bc | 5.52 b | 52.1 h | 235 bc | 72 c | 74.8 ef | 23.3 cde |
|  | L0S3 | L0 | S3 | 4.11 a | 6.65 a | 53.1 gh | 281 a | 83 a | 69.7 g | 21.9 f |
|  | L1S0 | L1 | S0 | 2.88 de | 4.19 d | 59.2 bc | 175 e | 68 c | 86.3 a | 24.5 ab |
|  | L1S1 | L1 | S1 | 2.85 e | 4.46 cd | 55.2 efg | 179 de | 70 c | 80.9 bcd | 24.0 abcd |
|  | L1S2 | L1 | S2 | 3.54 b | 5.47 b | 55.7 ef | 234 bc | 73 c | 77.5 de | 23.1 de |
|  | L1S3 | L1 | S3 | 4.43 a | 7.01 a | 54.3 fgh | 283 a | 82 a | 72.6 fg | 22.7 ef |
|  | L2S0 | L2 | S0 | 2.14 f | 2.92 e | 62.9 a | 132 f | 71 c | 79.9 cd | 24.8 a |
|  | L2S1 | L2 | S1 | 2.11 f | 2.99 e | 60.9 ab | 122 f | 74 c | 83.2 abc | 24.5 ab |
|  | L2S2 | L2 | S2 | 3.40 bcd | 4.93 bcd | 59.3 bc | 185 de | 81 ab | 82.0 bc | 23.8 abcde |
|  | L2S3 | L2 | S3 | 4.42 a | 6.70 a | 56.7 de | 253 ab | 83 a | 79.7 cd | 22.8 ef |
|  | **Mean** |  |  | **3.31 A** | **5.04 A** | **57.0 A** | **206 A** | **75 B** | **79.4 A** | **23.6 AB** |
| D3 | L0S0 | L0 | S0 | 2.77 ab | 4.28 d | 55.6 ab | 143 e | 82 ab | 85.0 a | 24.1 bc |
|  | L0S1 | L0 | S1 | 3.14 ab | 4.82 bcd | 56.3 a | 174 bcd | 79 ab | 81.4 abcd | 24.3 bc |
|  | L0S2 | L0 | S2 | 3.04 ab | 5.34 abc | 49.0 efg | 195 b | 80 ab | 72.9 ef | 23.0 bc |
|  | L0S3 | L0 | S3 | 3.23 ab | 5.92 a | 47.0 g | 233 a | 80 ab | 67.1 g | 22.2 c |
|  | L1S0 | L1 | S0 | 2.87 ab | 4.28 d | 57.7 a | 148 de | 74 b | 84.8 a | 27.2 a |
|  | L1S1 | L1 | S1 | 2.95 ab | 4.81 bcd | 52.9 bcd | 164 cde | 80 ab | 81.5 abcd | 23.7 bc |
|  | L1S2 | L1 | S2 | 2.83 ab | 4.70 bcd | 51.6 cde | 157 de | 85 a | 78.8 cd | 23.2 bc |
|  | L1S3 | L1 | S3 | 3.04 ab | 5.46 ab | 47.9 fg | 202 b | 83 ab | 69.2 fg | 22.8 bc |
|  | L2S0 | L2 | S0 | 2.72 b | 4.08 d | 57.2 a | 142 e | 83 a | 79.1 bcd | 25.0 ab |
|  | L2S1 | L2 | S1 | 2.91 ab | 4.41 d | 56.9 a | 144 e | 86 a | 83.4 ab | 24.5 bc |
|  | L2S2 | L2 | S2 | 2.86 ab | 4.50 cd | 54.8 abc | 142 e | 87 a | 83.4 abc | 23.9 bc |
|  | L2S3 | L2 | S3 | 3.29 a | 5.56 ab | 50.9 def | 188 bc | 85 a | 76.9 de | 23.0 bc |
|  | **Mean** |  |  | **2.97 B** | **4.85 A** | **53.1 B** | **169 B** | **82 A** | **78.6 A** | **23.9 A** |
| D4 | L0S0 | L0 | S0 | 1.51 cde | 2.58 bcd | 50.6 a | 95 bcde | 75 d | 78.2 a | 23.9 b |
|  | L0S1 | L0 | S1 | 1.34 def | 2.50 bcd | 47.5 ab | 80 def | 78 cd | 79.2 a | 23.8 b |
|  | L0S2 | L0 | S2 | 1.63 cd | 3.25 b | 42.8 cd | 118 ab | 77 d | 68.3 bc | 22.2 de |
|  | L0S3 | L0 | S3 | 1.32 def | 2.81 bc | 40.4 d | 111 abcd | 76 d | 62.4 c | 21.6 e |
|  | L1S0 | L1 | S0 | 1.49 cde | 2.52 bcd | 50.9 a | 85 cdef | 79 cd | 78.3 a | 24.1 b |
|  | L1S1 | L1 | S1 | 1.82 cd | 3.40 b | 45.7 bc | 118 ab | 75 d | 80.3 a | 23.5 bc |
|  | L1S2 | L1 | S2 | 1.48 cde | 2.76 bc | 46.0 bc | 93 bcde | 79 cd | 75.6 ab | 22.9 cd |
|  | L1S3 | L1 | S3 | 0.97 ef | 2.01 cd | 41.6 d | 78 ef | 78 cd | 61.3 c | 22.3 de |
|  | L2S0 | L2 | S0 | 2.46 ab | 4.48 a | 47.2 ab | 122 ab | 100 a | 68.2 bc | 25.4 a |
|  | L2S1 | L2 | S1 | 2.83 ab | 4.83 a | 50.4 a | 128 a | 95 ab | 80.2 a | 24.9 a |
|  | L2S2 | L2 | S2 | 1.96 bc | 3.35 b | 50.4 a | 99 abcde | 90 ab | 80.0 a | 23.9 b |
|  | L2S3 | L2 | S3 | 0.83 f | 1.71 d | 41.5 d | 54 f | 87 bc | 64.8 c | 22.7 d |
|  | **Mean** |  |  | **1.63 C** | **3.01 B** | **46.2 C** | **98 C** | **83 A** | **73.1 B** | **23.4 B** |
| ANOVA | | | N | *** | *** | *** | *** | ** | *** | ns |
|  |  |  | L | *** | ns | ns | *** | *** | *** | * |
|  |  |  | L*N | * | *** | ** | ** | *** | ns | ns |
|  |  |  | S | *** | *** | *** | *** | ns | *** | *** |
|  |  |  | S*N | * | *** | *** | *** | ** | ** | ns |
|  |  |  | L*S | *** | ns | ns | ns | ns | *** | ns |
|  |  |  | L*S*N | ns | *** | *** | ** | ns | ns | ns |

Note: The treatments of leaf-cutting and spikelet-thinning were carried out at full heading stage of the main crop. L0S0: no leaf-cutting and no spikelet-thinning; L0S1: no leaf-cutting and thinning 1/4 spikelets; L0S2: no leaf-cutting and thinning one half of spikelets; L0S3: no leaf-cutting and thinning 3/4 spikelets; L1S0: cutting the first leaf from the top and no spikelet-thinning; L1S1: cutting the first leaf from the top and thinning 1/4 spikelets; L1S2: cutting the first leaf from the top and thinning one half of spikelets; L1S3: cutting the first leaf from the top and thinning 3/4 spikelets; L2S0: cutting top three leaves and no spikelet-thinning; L2S1: cutting top three leaves and thinning 1/4 spikelets; L2S2: cutting top three leaves and thinning one half of spikelets; L2S3: cutting top three leaves and thinning3/4 spikelets. Means followed by different lower-case letters at the same node and different upper-case letters within one column denote signiﬁcantly different at the 0.05 level. D2, D3, and D4 represent the second node from the top, the third node from the top, and lower nodes below the third node of the stem, respectively. *, ** and *** represents the significant difference at the 5%, 1% and 0.1% level according to LSD test, respectively. ns represents no significant difference.

| Node | Treatment | Leaf-  cutting treatment | Spikelet-  thinning treatment | TZ9G (ng g^-1^) | TZR (ng g^-1^) | TZ (ng g^-1^) | IP9G (ng g^-1^) | IP (ng g^-1^) | IPR (ng g^-1^) |
| --- | --- | --- | --- | --- | --- | --- | --- | --- | --- |
| D2 | L0S0 | L0 | S0 | 34.842 c | 0.781 c | 2.959 bc | 0.412 c | 0.616 c | 1.280 a |
|  | L0S2 | L0 | S2 | 62.902 a | 0.927 a | 3.470 a | 0.770 a | 0.860 a | 0.975 b |
|  | L1S0 | L1 | S0 | 23.232 d | 0.760 c | 3.182 b | 0.434 c | 0.640 c | 0.627 e |
|  | L1S2 | L1 | S2 | 37.663 b | 0.814 bc | 2.910 c | 0.840 a | 0.631 c | 0.772 d |
|  | L2S0 | L2 | S0 | 14.738 e | 0.755 c | 3.171 bc | 0.473 c | 0.759 b | 0.805 d |
|  | L2S2 | L2 | S2 | 35.110 bc | 0.850 b | 3.732 a | 0.658 b | 0.877 a | 0.931 c |
|  | **Mean** |  |  | **34.748 C** | **0.815 B** | **3.238 C** | **0.598 B** | **0.730 B** | **0.898 A** |
| D3 | L0S0 | L0 | S0 | 60.235 b | 2.086 a | 9.515 a | 0.426 d | 1.926 a | 1.182 a |
|  | L0S2 | L0 | S2 | 68.424 a | 0.938 bc | 3.529 c | 0.917 a | 0.668 b | 0.832 b |
|  | L1S0 | L1 | S0 | 50.428 d | 1.145 b | 4.288 b | 0.444 d | 0.673 b | 0.733 c |
|  | L1S2 | L1 | S2 | 56.990 c | 0.915 c | 3.856 bc | 0.634 c | 0.548 c | 0.491 de |
|  | L2S0 | L2 | S0 | 40.967 f | 0.768 c | 3.529 c | 0.381 d | 0.562 c | 0.429 e |
|  | L2S2 | L2 | S2 | 45.692 e | 0.855 c | 3.474 c | 0.809 b | 0.675 b | 0.579 fg |
|  | **Mean** |  |  | **53.789 B** | **1.118 A** | **4.698 A** | **0.602 B** | **0.842 A** | **0.707 B** |
| D4 | L0S0 | L0 | S0 | 69.868 b | 0.691 d | 3.748 c | 0.842 b | 0.488 b | 0.447 cd |
|  | L0S2 | L0 | S2 | 75.956 a | 0.536 e | 2.469 e | 1.277 a | 0.661 a | 0.484 bc |
|  | L1S0 | L1 | S0 | 52.999 d | 0.767 c | 4.584 b | 0.634 c | 0.494 b | 0.521 b |
|  | L1S2 | L1 | S2 | 65.238 c | 0.628 d | 3.234 d | 0.855 b | 0.503 b | 0.394 d |
|  | L2S0 | L2 | S0 | 37.119 f | 1.340 a | 5.298 a | 0.572 c | 0.532 b | 0.684 a |
|  | L2S2 | L2 | S2 | 47.773 e | 0.945 b | 3.837 c | 0.808 b | 0.568 b | 0.255 e |
|  | **Mean** |  |  | **58.214 A** | **0.818 B** | **3.862 B** | **0.831 A** | **0.541 C** | **0.464 C** |
|  | ANOVA |  | N | *** | ** | *** | *** | ** | *** |
|  |  |  | L | ** | * | ** | *** | *** | *** |
|  |  |  | S | *** | *** | *** | *** | *** | *** |
|  |  |  | L*N | *** | *** | *** | *** | *** | *** |
|  |  |  | S*N | *** | *** | *** | ns | *** | *** |
|  |  |  | L*S | * | *** | *** | ns | *** | *** |
|  |  |  | L*S*N | *** | *** | *** | ns | *** | *** |

**Supplemental Table 4.** Content of six cytokinins in stems and buds at each node 15 days after leaf-cutting and spikelets-thinning treatment in 2018

Note: L0: represent the treatment of no leaf-cutting; L1: represent the treatment of cutting top of one leaf; L2: represent the treatment of cutting top of three leaves; S0 and S2 represent the treatment of thinning 0 and 1/2 spikelets, respectively. D2, D3 and D4 represent the second, third and lower than third nodes from the top, respectively. Data followed by different lower-case letters in the same year and different upper-case letters within one column denote significant differences between treatments at the 5 % level according to LSD test. ***, ** and * represents the significant difference at the 0.1 %, 1% and 5% level according to LSD test, respectively. ns represent no significant difference.

**Supplemental Table 5.** Contents of six cytokinins in stems and buds at each node 15 days after leaf-cutting and spikelets-thinning treatment in 2019

| Node | Treatment | Leaf cutting  treatment | Spikelet- thinning treatment | TZ9G (ng g^-1^) | TZR (ng g^-1^) | TZ (ng g^-1^) | IP9G (ng g^-1^) | IP (ng g^-1^) | IPR (ng g^-1^) |
| --- | --- | --- | --- | --- | --- | --- | --- | --- | --- |
| D2 | L0S0 | L0 | S0 | 23.71 fg | 0.710 bc | 3.000 b | 0.415 g | 0.554 bc | 0.641 cde |
|  | L0S1 | L0 | S1 | 52.44 d | 0.702 bc | 2.841 b | 0.924 de | 0.650 abc | 0.699 cd |
|  | L0S2 | L0 | S2 | 61.46 c | 0.708 bc | 2.757 b | 1.016 cd | 0.642 abc | 0.842 bc |
|  | L0S3 | L0 | S3 | 108.58 a | 0.829 b | 2.849 b | 1.877 a | 0.667 abc | 0.882 bc |
|  | L1S0 | L1 | S0 | 16.37 g | 0.110 e | 1.109 d | 0.592 fg | 0.546 bc | 0.344 f |
|  | L1S1 | L1 | S1 | 27.28 ef | 0.116 e | 1.074 d | 1.052 bcd | 0.572 bc | 0.457 def |
|  | L1S2 | L1 | S2 | 57.76 cd | 0.394 d | 1.621 c | 1.083 bcd | 0.701 ab | 1.012 b |
|  | L1S3 | L1 | S3 | 84.49 b | 1.158 a | 3.698 a | 1.160 bc | 0.784 a | 1.295 a |
|  | L2S0 | L2 | S0 | 6.85 h | 0.140 e | 0.978 d | 0.775 ef | 0.522 c | 0.321 f |
|  | L2S1 | L2 | S1 | 7.46 h | 0.144 e | 1.083 d | 0.961 cde | 0.523 c | 0.377 ef |
|  | L2S2 | L2 | S2 | 32.15 e | 1.199 a | 3.744 a | 1.087 bcd | 0.564 bc | 1.380 a |
|  | L2S3 | L2 | S3 | 59.18 cd | 0.645 c | 2.755 b | 1.275 b | 0.679 abc | 0.896 bc |
|  | **Mean** |  |  | **44.810 C** | **0.571 B** | **2.292 C** | **1.018 B** | **0.617 A** | **0.762 A** |
| D3 | L0S0 | L0 | S0 | 61.451 de  51 de | 0.979 a | 3.879 a | 0.297 e | 0.536 e | 0.528 cd |
|  | L0S1 | L0 | S1 | 70.492 cd | 1.031 a | 3.987 a | 0.598 d | 0.676 bc | 0.515 d |
|  | L0S2 | L0 | S2 | 71.381 c | 0.755 bc | 3.349 b | 0.633 cd | 0.629 bcd | 0.398 ef |
|  | L0S3 | L0 | S3 | 89.279 a | 0.839 ab | 3.697 ab | 1.054 b | 0.673 bc | 0.620 bc |
|  | L1S0 | L1 | S0 | 66.700 cde | 0.570 cdef | 2.006 de | 0.602 d | 0.527 e | 0.686 b |
|  | L1S1 | L1 | S1 | 67.418 cde | 0.673 bcd | 2.678 c | 0.664 cd | 0.592 cde | 0.513 d |
|  | L1S2 | L1 | S2 | 75.431 bc | 0.542 def | 2.350 cde | 0.664 cd | 0.790 a | 0.664 b |
|  | L1S3 | L1 | S3 | 81.699 ab | 0.613 def | 2.417 cd | 1.025 b | 0.596 cde | 0.965 a |
|  | L2S0 | L2 | S0 | 30.361 f | 0.575 cdef | 2.422 cd | 0.637 cd | 0.554 de | 0.469 de |
|  | L2S1 | L2 | S1 | 34.404 f | 0.458 ef | 2.175 de | 0.653 cd | 0.562 de | 0.355 f |
|  | L2S2 | L2 | S2 | 36.548 f | 0.369 f | 2.008 de | 0.692 cd | 0.703 b | 0.530 cd |
|  | L2S3 | L2 | S3 | 58.109 e | 0.476 dfg | 1.913 e | 0.903 bc | 0.594 cde | 0.505 d |
|  | **Mean** |  |  | **61.939 B** | **0.657 A** | **2.740 B** | **0.788 C** | **0.619 A** | **0.562 B** |
| D4 | L0S0 | L0 | S0 | 74.52 fg | 0.950 a | 4.388 ab | 1.487 c | 0.639 ab | 0.407 bc |
|  | L0S1 | L0 | S1 | 87.14 de | 0.764 bc | 4.201 ab | 1.461 c | 0.542 abc | 0.454 b |
|  | L0S2 | L0 | S2 | 87.38 de | 0.742 bc | 3.918 bc | 1.486 c | 0.492 c | 0.361 bc |
|  | L0S3 | L0 | S3 | 106.66 ab | 0.697 c | 3.454 cd | 2.221 b | 0.631 ab | 0.459 b |
|  | L1S0 | L1 | S0 | 68.38 g | 0.867 ab | 4.492 a | 1.288 de | 0.589 abc | 0.448 b |
|  | L1S1 | L1 | S1 | 81.46 ef | 0.749 bc | 2.732 ef | 1.214 e | 0.492 c | 0.383 bc |
|  | L1S2 | L1 | S2 | 104.17 b | 0.724 c | 2.627 ef | 1.236 e | 0.531 bc | 1.064 a |
|  | L1S3 | L1 | S3 | 114.22 a | 0.707 c | 2.536 ef | 1.591 c | 0.591 abc | 1.090 a |
|  | L2S0 | L2 | S0 | 70.17 g | 0.551 d | 3.003 de | 0.781 f | 0.557 abc | 0.116 d |
|  | L2S1 | L2 | S1 | 86.80 de | 0.465 de | 2.421 f | 1.440 cd | 0.648 a | 0.285 c |
|  | L2S2 | L2 | S2 | 92.62 cd | 0.438 de | 2.305 f | 2.623 a | 0.640 ab | 0.368 bc |
|  | L2S3 | L2 | S3 | 98.49 bc | 0.354 e | 1.562 g | 1.590 c | 0.490 c | 0.485 b |
|  | **Mean** |  |  | **89.334 A** | **0.667 A** | **3.137 A** | **1.535 A** | **0.570 A** | **0.493 C** |
| ANOVA | | | N | *** | * | ** | *** | ns | *** |
|  |  |  | L | *** | *** | *** | ns | ns | *** |
|  |  |  | S | *** | *** | * | *** | ** | *** |
|  |  |  | L*N | *** | *** | *** | *** | ns | *** |
|  |  |  | S*N | *** | *** | *** | *** | ** | *** |
|  |  |  | L*S | *** | *** | *** | *** | ns | *** |
|  |  |  | L*S*N | ** | *** | *** | *** | ns | *** |

Note: L0: represent the treatment of no leaf-cutting; L1: represent the treatment of cutting top of one leaf; L2: represent the treatment of cutting top of three leaves; S0, S1, S2 and S3 represent the treatment of thinning 0, 1/4, 1/2 and 3/4 spikelets, respectively. D2, D3 and D4 represent the second, third and lower than third nodes from the top, respectively. Data followed by different lower-case letters in the same year and different upper-case letters within one column denote significant differences between treatments at the 5 % level according to LSD test. ***, ** and * represents the significant difference at the 0.1 %, 1% and 5% level according to LSD test, respectively. ns represent no significant difference.

**Supplemental Table 6.** ANOVA analysis of leaf-cutting treatment and spikelet-thinning treatment on the single length and number of regenerated buds at separate node in 2018 and 2019

| Year | Factor | D2 |  |  | D3 |  |  | D4 |  |
| --- | --- | --- | --- | --- | --- | --- | --- | --- | --- |
|  |  | SBL (cm) | BN (no m^2^) |  | SBL (cm) | BN (no m^2^) |  | SBL (cm) | BN (no m^2^) |
| 2018 | L | *** | *** |  | *** | *** |  | *** | *** |
|  | S | *** | *** |  | *** | *** |  | *** | *** |
|  | L*S | * | ns |  | ** | ns |  | ** | ns |
|  |  |  |  |  |  |  |  |  |  |
| 2019 | L | * | ** |  | ** | ** |  | *** | *** |
|  | S | *** | *** |  | *** | *** |  | *** | *** |
|  | L*S | ns | ns |  | ns | ns |  | * | *** |

Note: L and S represent the treatment of leaf-cutting and spikelet-thinning, respectively. ***, ** and * represents the significant difference at the 0.1 %, 1 % and 5% level according to LSD test, respectively. ns represents no significant difference.
